# MicroRNA-188-5p targeting Forkhead Box L1 promotes colorectal cancer progression via activating Wnt/β-catenin signaling

**DOI:** 10.32604/or.2022.03178

**Published:** 2022-07-13

**Authors:** JIALIN WU, ZEHONG CHEN, WENWEI LIU, YONGXIN ZHANG, WEI FENG, YUJIE YUAN, JINNING YE, LIANG WANG, SHIRONG CAI, YULONG HE, SUIJING WU, WU SONG

**Affiliations:** 1 Center of Gastrointestinal Surgery, The First Affiliated Hospital, Sun Yat-sen University, Guangzhou, China; 2 Department of Hepatobiliary and Pancreatic Surgery, Union Shenzhen Hospital, Huazhong University of Science and Technology, Shenzhen, China; 3 Department of Gastrointestinal Surgery, The Third Affiliated Hospital, Sun Yat-sen University, Guangzhou, China; 4 Department of Hematology, Guangdong General Hospital, Guangzhou, China

**Keywords:** miR-188, Colorectal adenocarcinoma, Oncogenesis, Oncologic progression, Wnt signaling

## Abstract

**Objective:**

MicroRNA-188-5p (miR-188) enhances oncologic progression in various human malignancies. This study aimed to explore its role in colorectal cancer (CRC).

**Materials and Methods:**

Human CRC tissues paired with normal tissues, and several CRC cell lines were utilized. Real-time quantitative PCR was applied to measure the expression of miR-188. Overexpression and knockdown were used to access the function of miR-188 and to investigate whether FOXL1/Wnt signaling mediates such function. The proliferation, migration and invasion of cancer cells were evaluated by CCK8, wound-healing and transwell assays, respectively. Whether FOXL1 acted as a direct target of miR-188 was verified by dual-luciferase reporter assays.

**Results:**

Levels of miR-188 were upregulated in CRC tissues than in paired-normal tissues, as well as in various CRC cell lines. High expression of miR-188 was strongly associated with advanced tumor stage, accompanied with significant tumor cell proliferation, invasion and migration. It was confirmed that FOXL1 played positive crosstalk between miR-188 regulation and downstream Wnt/β-catenin signaling activation.

**Conclusions:**

All findings indicate that miR-188 promotes CRC cell proliferation and invasion through targeting FOXL1/Wnt signaling and could be served as a potential therapeutic target for human CRC in the future.

## Introduction

Known as one of the most common types of diagnosed cancer, colorectal cancer (CRC) is the fourth leading cause of cancer-associated mortalities worldwide [1,2]. Although strategies to manage CRC have significantly improved in recent years, the prognosis of advanced CRC remains poor owing to local migration and distant metastasis [3]. Therefore, it is crucial to elucidate the mechanisms underlying tumorigenesis and explore more effective therapeutic targets of CRC.

An increasing number of studies have demonstrated that micro (mi) RNAs, a family of small non-coding RNAs, mainly function by binding to the 3’-untranslated region (UTR) of target genes. This can then cause degradation or translational repression, and when aberrantly expressed, is associated with various physiological or pathological processes in multiple types of human cancer including CRC [4–8]. Previous studies also showed that aberrantly expressed miRNAs are associated with the proliferation, apoptosis, and metastasis of cancer cells [9].

MiRNA-188-5p (miR-188) is regulated aberrantly in diverse types of diseases such as hepatocellular carcinoma, prostate cancer, and leukemia, which indicates it may serve as a tumorigenesis factor. Fang et al. found that miR-188 was downregulated in hepatocellular carcinoma, and suppressed tumor cell proliferation and metastasis by directly targeting FGF5 [10]. Zhang et al. discovered that miR-188 inhibited tumor growth and metastasis in prostate cancer by repressing LAPTM4B expression [11]. However, only some studies illustrated the correlation between miR-188 and the progression of CRC, and the precise biological roles of miR-188 are largely illusive.

Forkhead Box L1 (FOXL1), a transcription factor gene located in 16q24.1, was initially discovered in the mesenchyme of the gastrointestinal tract and reported to be expressed ectopically in some types of carcinomas [12]. FOXL1 acts as a tumor suppressor and an outcome predictor in pancreatic cancer, clear cell renal cell carcinoma, and osteosarcoma [13–15]. In the gastrointestinal tract, FOXL1 might closely associate with the Wnt/β-catenin signaling pathway, a pathway that plays a critical role in regulating gastrointestinal cell proliferation and mutations, as well as tumorigenesis [12].

In the present study, we demonstrated that miR-188 was upregulated in CRC tissues and cell lines. Moreover, the expression of miR-188 was upregulated in advanced CRC when compared with early CRC. MiR-188 promoted CRC cell proliferation, invasion, and migration. We also firstly identified that FOXL1 was a direct target of miR-188. This involved increased miR-188 promoting the proliferation, migration, and invasion of CRC cells. Notably, knockdown of FOXL1 upregulated Wnt signaling in CRC cells while inhibition of miR-188 remarkably reversed this process. These findings suggest that miR-188 promotes CRC cell proliferation and invasion through targeting FOXL1/Wnt signaling. This provides new insights into the biological function of miR-188 and may assist in developing it as a potential therapeutic target for CRC.

## Materials and Methods

### Patient samples

A total of 46 patients with confirmed diagnosis of CRC was included for this study. Fresh-paired samples, including partial tumor and adjacent normal tissues, were collected from resected specimens in the theatre during the study period. All collected samples were frozen immediately in liquid nitrogen and stored at −80°C until future tests. The ethical approval for human subjects was obtained from the IRB of our hospital in 2017, with written informed consent obtained from involved patients before surgery.

### Cell lines and culture

Human CRC cell lines including HCT116, SW480, LS174T, LOVO, HT29, and SW620 were obtained from the American Type Culture Collection (ATCC, Manassas, VA, USA). The normal human colon cells familial hypercholesterolemia (FHC) was purchased from the Shanghai Cell Bank of the Chinese Academy of Science (Shanghai, P.R. China). FHC and HCT116 cells were cultured in RPMI 1640 (Gibco, San Jose, CA, USA) while SW480, LS174T, LOVO, HT29, and SW620 cells were cultivated in Dulbecco’s modified Eagle’s medium (DMEM, Gibco, Grand Island, NY) supplemented with 10% fetal bovine serum (Gibco) and 0.1% penicillin/streptomycin (Invitrogen, Carlsbad, CA, USA). The cell incubator was humidified and maintained at 37°C with 5% CO_2_.

### RNA extraction and quantitative real-time polymerase chain reaction (qRT-PCR) analysis

Total RNA of clinical specimens and cell lines was extracted using Trizol reagent (Invitrogen). qRT-PCR was conducted by a standard SYBR Green PCR kit (TaKaRa, Dalian, China) on the LightCycler 480 platform (Roche, Meylan, France). The gene expression data of both mRNA and lncRNA were normalized to the geometric mean of the glyceraldehyde-3-phosphate dehydrogenase (GAPDH) housekeeping gene to control for variability in expression levels, while miRNAs were normalized to U6. The specific mRNA primers are presented in Table 1. Data were collected and analyzed using 2−ΔΔCt method.

**Table 1:** Specific primers for reverse transcription-quantitative PCR used in the current study

Gene	Sequence(5’→3’)	
	Forward	Reverse
FOXL1	GCCTCGCCCATGCTGTATC	CGTTGAGCGTGACCCTCTG
β-catenin	ATGTCCAGCGTTTGGCTGAA	TGGTCCTCGTCATTTAGCAGTT
c-MYC	GTCTGTGCATTTCTGGTTGCA	TTTCTAGACTTTCATGTTTGTCTTTTTGTC
Cyclin D1	GTCAAGAGGCGAACACACAAC	TTGGACGGACAGGATGTATGC
GAPDH	GCTCTCTGCTCCTCCTGTTC	ACGACCAAATCCGTTGACTC

### Cell transfection

The RiboFect CP Transfection Kit (Ribobio, Guangzhou, China) was used for transfection according to the product description. MiRNA-188 mimic (miR-188), miR-188 mimic control (miR-NC), miR-188 inhibitor (in-miR-188), and inhibitor control (in-miR-NC) were purchased from Ribobio. FOXL1 small interfering (si)RNA (siRNA-FOXL1) and scrambled siRNA (siRNA-NC), as well as pcDNA3.1-FOXL1 (pcDNA-FOXL1) and pcDNA3.1-control (pcDNA- NC), were obtained from Igenebio (Guangzhou, China).

### Wound-healing assay

About 3 × 10^5^ cells transfected with miRNA, siRNA, or pcDNA3.1, and their controls, were plated on six-well plates until the cells grew into monolayers. The cell layer was then scratched with a sterile plastic tip and washed with phosphate-buffered saline three times. The cells were cultured in DMEM or RPMI 1640 containing 1% fetal bovine serum. Images of the cells were acquired at 0 and 48 h after the scratch under a microscope. The distance between the two edges of the scratch was measured using the Digimizer software system (www.digimizer.com).

### Cell proliferation assays

Cell Counting Kit 8 (CCK8) (Dojindo, Kumamoto, Japan) was used to measure cell viability. Cells transfected with miRNA, siRNA, or pcDNA3.1 were grown on 96-well plates. Cell viability was monitored every 24 h according to the manufacturer’s protocol.

### Cell migration and invasion assays

The cell migration and invasion assays were performed using Transwell units with an 8-μm pore size (BD Biosciences, Franklin Lakes, NJ, USA). For migration assays, 5 × 10^4^ cells were placed into the upper chamber with serum-free medium. For invasion assays, 5 × 10^4^ cells were plated into the upper chamber which were precoated with Matrigel in serum-free medium (BD Biosciences). The lower chamber contained DMEM or RPMI 1640 with 10% fetal bovine serum. After incubation for 24 h at 37°C, cells in the upper chamber were removed. Cells that transferred to the lower membrane surface were fixed with methanol and stained with 1.0% crystal violet. Cells on the lower chamber membrane were photographed by using an inverted microscope and counted manually.

### Western blotting

Total cell protein was extracted using the Total Protein Extraction Reagent Kit (KeyGEN, Jiangsu, China). Protein concentrations were monitored using a BCA Protein Assay Kit (KeyGEN). A total of 30 µg protein was separated using 10% sodium dodecyl sulfate-polyacrylamide gel electrophoresis and then transferred onto a polyvinylidene difluoride membrane (EMD Millipore, Billerica, MA, USA). The membranes were cut into pieces according to the molecular weight of each protein after being blocked with 5% non-fat milk. Then, the membranes were incubated with primary antibodies against GAPDH (rabbit monoclonal), β-catenin, c-Myc, or cyclin D1 (1:2000, Abcam, Cambridge, UK) overnight at 4°C. The membranes were then incubated with a secondary antibody (1:5000, Biogot Technology, Nanjing, China) for 2 h at room temperature. An enhanced chemiluminescence substrate (EMD Millipore) was used to visualize the protein bands.

### Immunohistochemistry staining

Paraformaldehyde-fixed paraffin sections were deparaffinized and rehydrated, then immersed in 10 mM citrate buffer for antigen retrieval. Endogenous peroxidases were blocked with 3% hydrogen peroxide. The sections were then blocked with 10% goat plasma for 30 min and the slides were incubated overnight at 4°C in a moist chamber with the primary antibody against FOXL1 (1:400, Abnova, Walnut, CA, USA). The universal horseradish peroxidase polymer was then added before incubation for 30 min in a moist chamber. Staining was performed using a 3,3’-diaminobenzidine solution (Biocare Medical, Pacheco, CA, USA), with hematoxylin used for counterstaining.

### Dual luciferase reporter assay

Binding sites between miR-188 and FOXL1 were predicted by bioinformatics tools (microRNA.org, Starbase v2.0, and miRcode). The fragment of the FOXL1 3’-UTR containing the predicted miR-188 binding or mutant sequences was synthesized then cloned into the psiCHECK-2 vector (Promega, Madison, WI, USA). Human HEK293 cells (ATCC) were co-transfected with the luciferase reporter vector with or without the miR-188 mimic. The Dual Luciferase Reporter Assay System (Promega) was applied to measure luciferase activity according to the manufacturer’s protocol. Results were normalized to Renilla luciferase activity.

### Statistical analyses

All statistical analyses were performed using SPSS 19.0 statistical software (IBM, Chicago, IL, USA) or GraphPad Prism (Version 7.0, GraphPad Software, La Jolla, CA, USA). Data are presented as means ± standard error of the mean (SEM). The statistical analysis approaches included Student’s *t*-test (2-tailed), one-way ANOVA, and the Mann–Whitney U test. *p* < 0.05 was considered statistically significant.

## Results

### MiR-188 expression was upregulated in CRC tissues and cell lines

**Figure 1:**
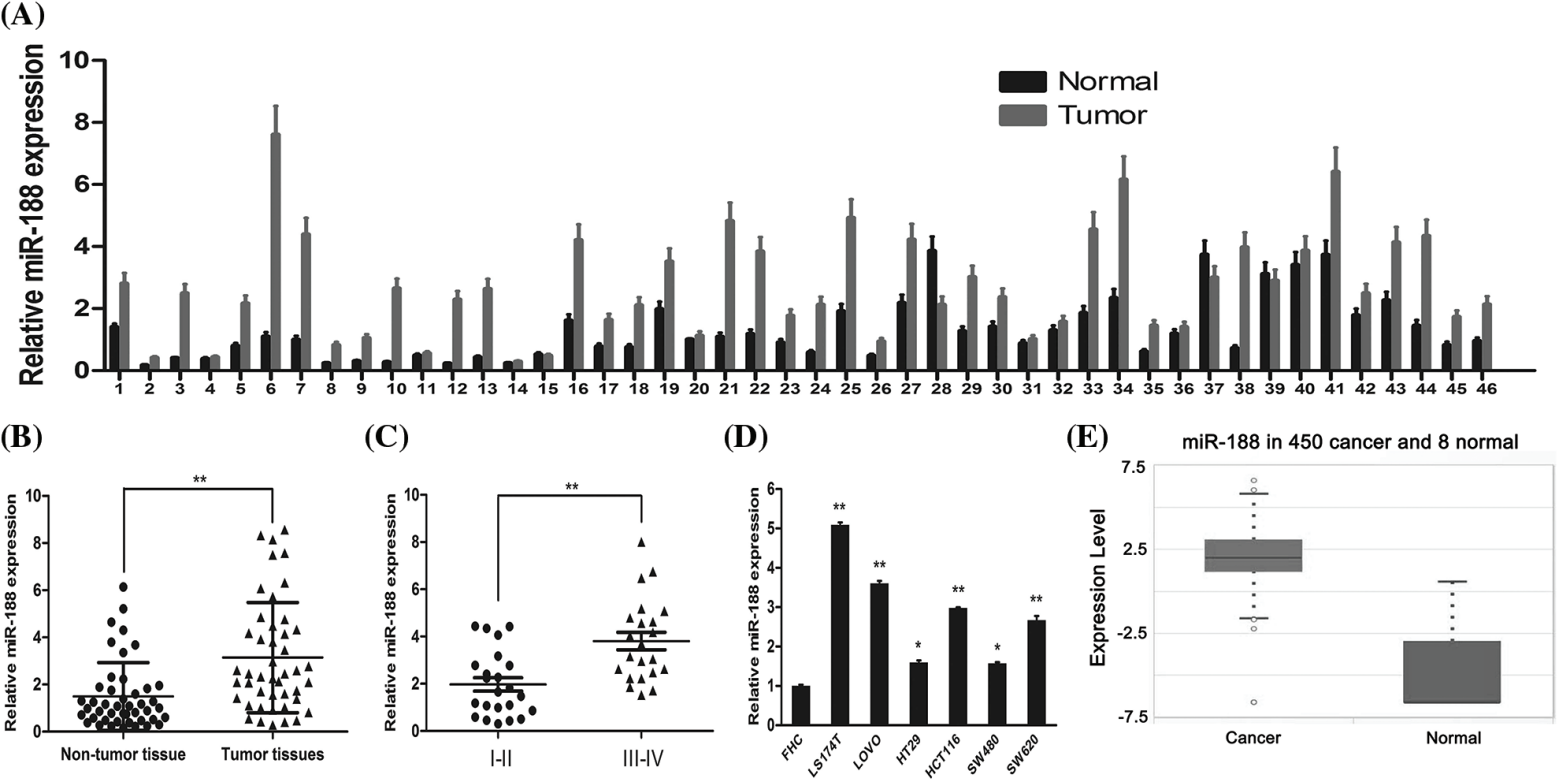
**Expression of miR-188 was upregulated in human colorectal cancer tissues and cell lines.** (A) Expression levels of miR-188 was verified by qRT-PCR in 46 pairs of human colon cancer tissues and adjacent normal tissues. (B) MiR-188 level was significantly upregulated in human colon cancer tissues compared with normal tissues. (C) Expression of miR-188 in patients of stage III–IV was remarkably higher than that of patients in stage I–II classified by NCCN TNM system. (D) The up-regulation of miR-188 in CRC was verified by using STARBASE 3.0. (E) The expression of miR-188 was significantly upregulated in colorectal cancer cell lines than normal colorectal mucous membrane (FHC). *p < 0.05, **p < 0.01.

To confirm the involvement of miR-188 in the development of CRC, the real-time qRT-PCR was performed to explore the expression level of miR-188 in human CRC and adjacent normal tissues (Fig. 1A). The results showed that the expression of miR-188 was significantly increased in CRC tissues when compared with adjacent normal tissues (Fig. 1B). The expression level of miR-188 in stage III–IV CRC tissues was significantly higher than that in stage I–II tissues (Fig. 1C). Moreover, the up-regulation of miR-188 was further verified in online dataset STARBASE 3.0 (Fig. 1D). Furthermore, miR-188 was upregulated in six CRC cell lines when compared with normal colorectal mucous membranes pooled from three healthy individuals (Fig. 1E).

### MiR-188 promoted CRC cell proliferation, migration, and invasion

**Figure 2:**
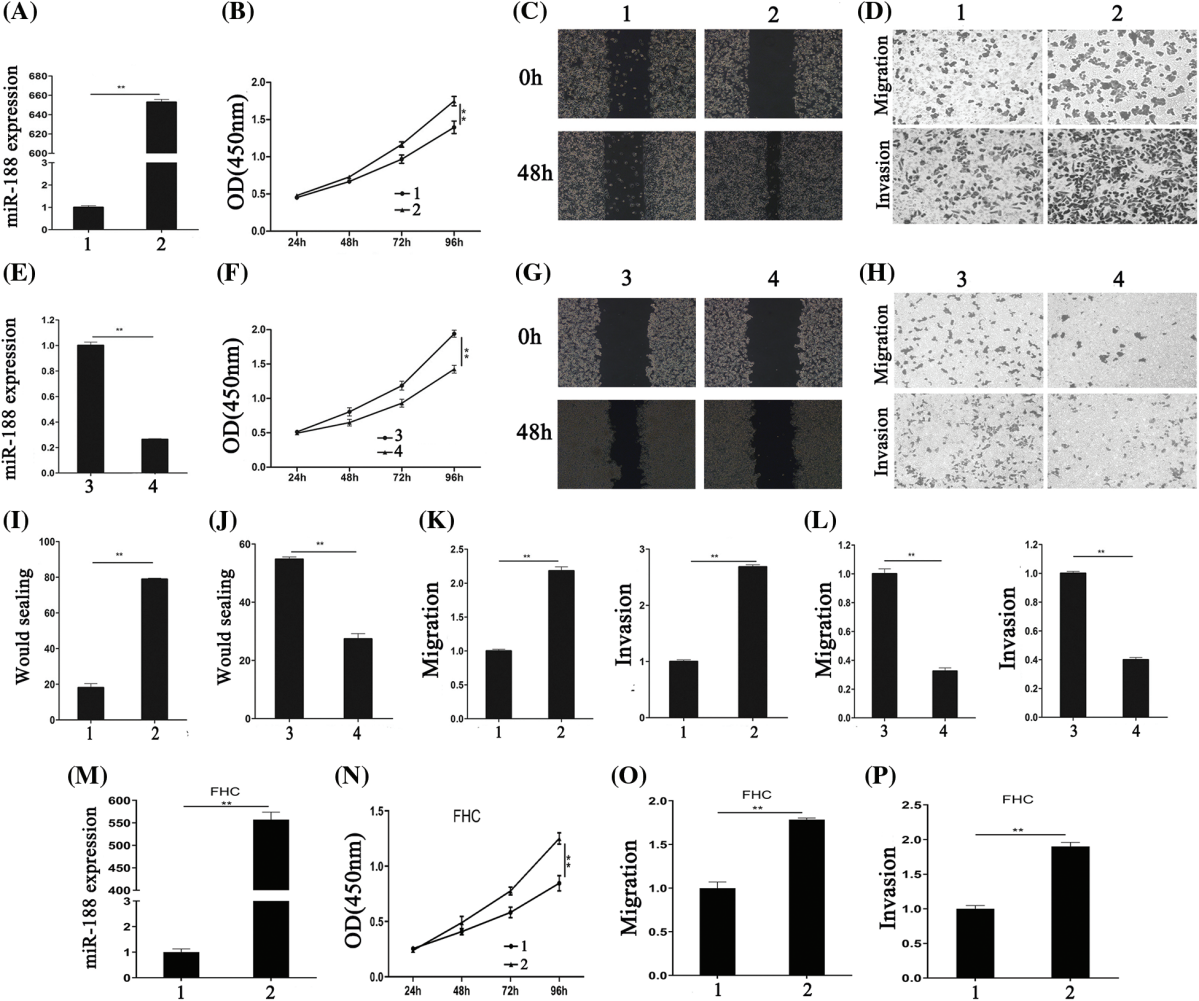
**Aberrant expression of miR-188 significantly regulated the proliferation, migration and invasion of colorectal cancer cells.** (A) The efficiency of miR-188 mimic was verified in SW480. (B) CCK8 assays showed that miR-188 promoted cell proliferation in SW480. (C&I) Wound healing assays were performed to investigate the migratory ability of SW480. (D&K) miR-188 significantly promoted migration and invasion in SW480. (E) The efficiency of miR-188 inhibitor was verified in LS174t. (F) Downregulation of miR-188 notably inhibited cell proliferation in LS174t. (G&J) The wound healing assays showed the proliferous and migratory ability of LS174t was weakened by miR-188 inhibitor. (H&L) Down-expression of miR-188 significantly inhibited migration and invasion in LS174t. (M) The over-expression of miR-188 in FHC was verified. (N) CCK8 assays indicated miR-188 promoted FHC proliferation. (O & P) miR-188 potently promoted the capabilities of migration and invasion in FHC.1: Mimic-ctr; 2: miR-188 mimic; 3: Inhibitor-ctr; 4: miR-188 inhibitor. **p < 0.01.

The biological functions of miR-188 in CRC, including cell proliferation, migration, and invasion, were assessed. SW480 cells were selected for transfection with an miR-188 mimic considering their low endogenous miR-188 expression, while LS174t cells were selected for transfection with an miR-188 inhibitor for their high endogenous miR-188 level. Control miRNA (miR-ctr) was also transfected into these cells. The efficiency of transfection with the miR-188 mimic was confirmed by qRT-PCR (Fig. 2A).

CCK8 and wound healing assays were performed to detect the effect of miR-188 on cell proliferation. The results of the CCK8 assay showed that miR-188 overexpression significantly promoted the proliferation of SW480 cells (Fig. 2B). This was consistent with the results of the wound healing assay (Figs. 2C and 2I).

To determine whether overexpression of miR-188 exhibited a crucial role in migration and invasion in CRC cells, Transwell migration (without Matrigel) and invasion (with Matrigel) assays were carried out in SW480 cells that were transfected with the miR-188 mimic. The results demonstrated that SW480 cells transfected with miR-188 exhibited higher migratory and invasive activities compared with the control group (Figs. 2D and 2K). The effect of downregulating miR-188 on CRC cells were also tested (Fig. 2E). The results showed that miR-188 knockdown significantly inhibited cell proliferation, and the migration and invasion capacities of LS174t cells (Figs. 2F–2H, 2J and 2L). Moreover, the normal human colon cells FHC were transfected with miR-188 mimic (Fig. 2M). CCK8 experiments showed that miR-188 mimic significantly inhibited FHC cell proliferation (Fig. 2N). Moreover, the capabilities of FHC in migration and invasion were also potently inhibited by miR-188 over-expression (Figs. 2O and 2P). Hence, these results indicated that miR-188 might act as tumor progression-factor in CRC.

### MiR-188 bound to the 3’UTR of FOXL1 directly

**Figure 3:**
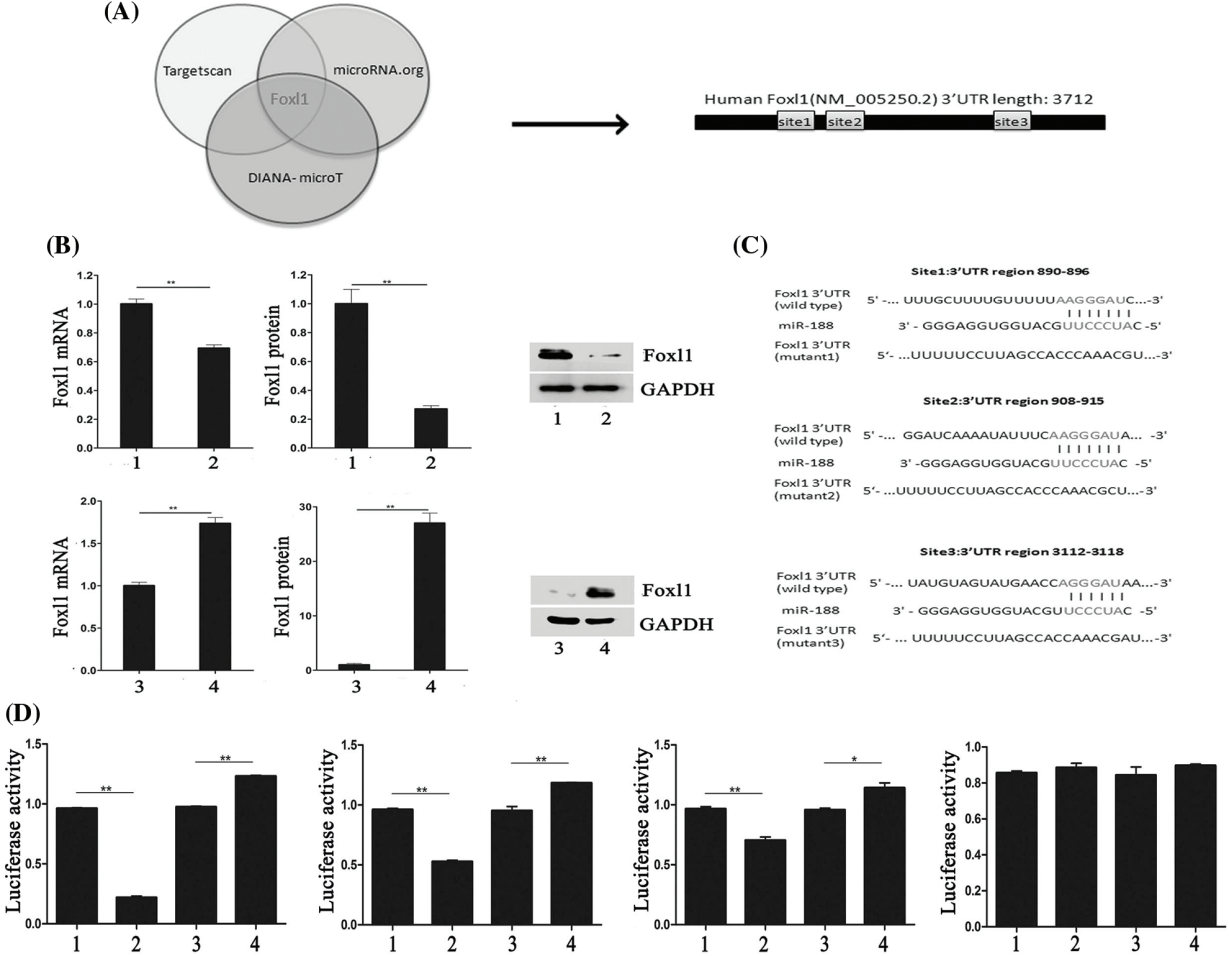
**MiR-188 promoted the function of tumorigenesis in colorectal cancer cells by directly targeting FOXL1.** (A) FOXL1 was predicted as a potential downstream target for miR-188. (B) Level of mRNA and protein of FOXL1 after upregulation and down-expression of miR-188 were identified. (C) Luciferase reporters containing three different potential binding sites were presented. (D) The results of luciferase reporter assays showed that miR-188 directly targeted 3’UTR of FOXL1 in 3112-3118 region. 1: mimic-ctr; 2: miR-188 mimic; 3: Inhibitor-ctr; 4: miR-188 inhibitor. *p < 0.05, **p < 0.01.

We investigated candidate targets for miR-188 by applying prediction algorithms provided by TargetScan, DIANA-microT, and microRNA.org. FOXL1 was predicted as a potential downstream target for miR-188 and its putative binding sites are presented in Fig. 3A. Moreover, we demonstrated the effect of miR-188 on FOXL1 in human CRC cells. The results of qRT-PCR and western blotting showed that the mRNA and protein levels of FOXL1 were consistently downregulated in SW480 cells transfected with the miR-188 mimic, while transfection with the miR-188 inhibitor led to the reverse in LS174t cells (Fig. 3B).

To explore whether FOXL1 was a direct target of miR-188 in the CRC cellular environment, dual-luciferase reporter assays were carried out. Luciferase reporters containing three different potential binding sites were generated (Fig. 3C). W To further determine the mechanism of interaction between miR-188 and FOXL1, dual luciferase reporter assays were conducted. The full length of wild type 3’UTR of FOXL1 was into reporter plasmids (Foxl1-wt). Mutant vectors containing three mutant sites were also constructed, respectively (mut1-Foxl1, mut2-Foxl1, and mut3-Foxl1). Wt-Foxl1 vector along with miR-188 mimic or miR-188 inhibitor or miR-Ctrl were then co-transfected into HEK293 cells for measuring luciferase activity. Mutant vectors (Foxl1-mt1, Foxl1-mt2, or Foxl1-mt3) were also respectively transfected into HEK293 cells with high expression of miR-188 or knockdown of miR-188. Results showed that compared to miRNA controls, overexpression of miR-188 induced a strong decline in the luciferase activity of Foxl1-wt, while knockdown of miR-188 exhibited a significantly increase (Fig. 3D). These effects were analogous in cells with Foxl1-mt1 vector or Foxl1-mt2 vector but not Foxl1-mt3 vector, whose luciferase activity could not be affected significantly by miR-188 mimic or miR-188 inhibitor(Fig. 3D). There results demonstrated that miR-188 bound to the 3’UTR of FOXL1, precisely, in the third predictive target site of 3112-3118 position of 3’UTR.

Immunohistochemistry was performed to explore the association between miR-188 and FOXL1 in CRC tissues. Patients were divided into high and low miR-188 groups according to the expression of this miRNA. Compared with low miR-188 tissues, the expression of FOXL1 was significantly increased in high miR-188 tissues (Fig. 6C). Taken together, we conclude that miR-188 promoted cell proliferation, migration, and invasion by directly targeting FOXL1 in CRC.

### The effects of miR-188 on CRC cells were rescued by FOXL1

**Figure 4:**
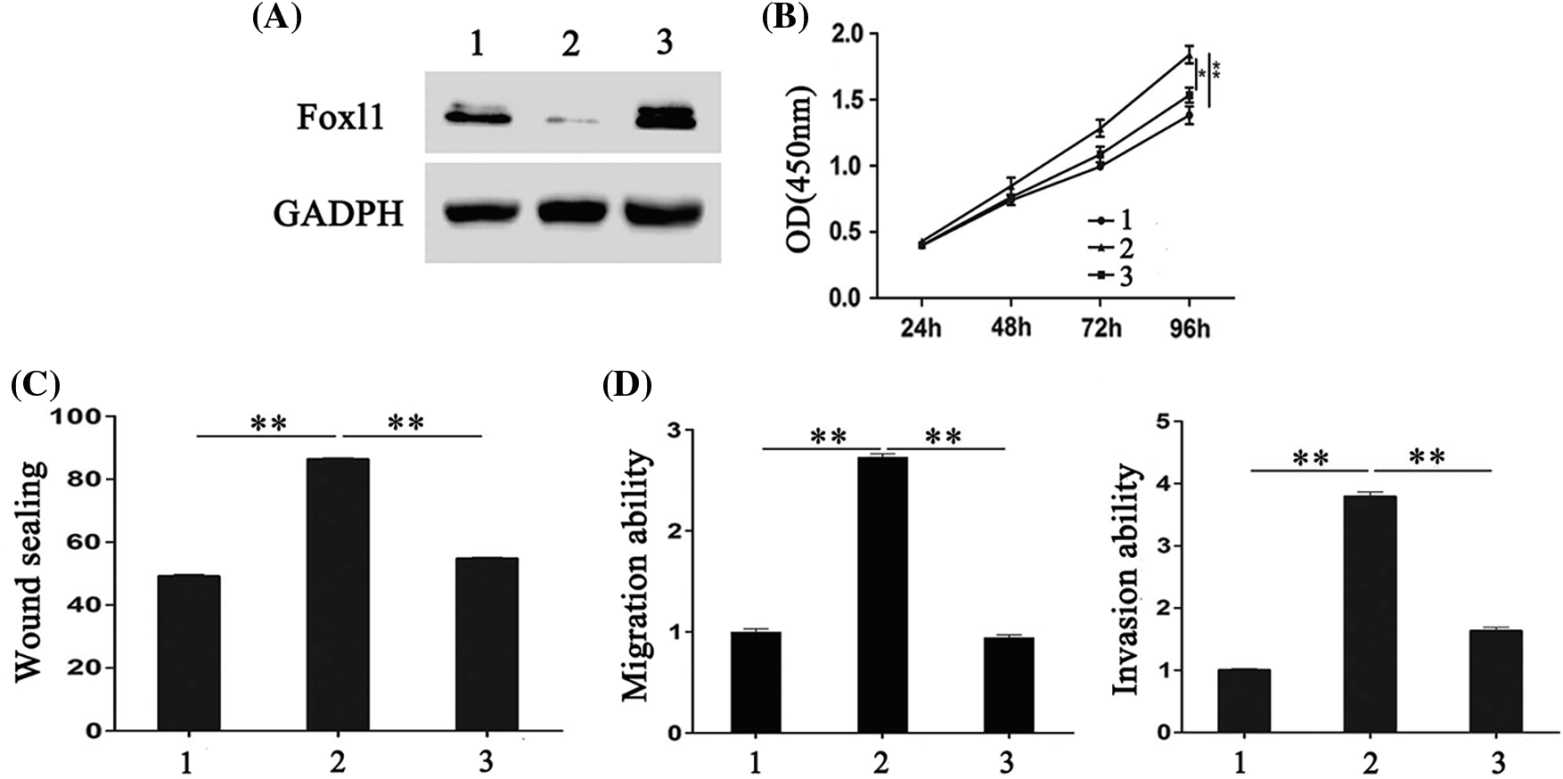
**FOXL1 mediated the stimulating effect of miR-188 on the proliferation, migration and invasion of colorectal cancer cells.** (A) The expression of FOXL1 protein in SW480 cells were detected after transfection with miR-188 mimic and FOXL1 pcDNA3.1. (B-D) Cell proliferation, migration and invasion of SW480 was assessed after transfection of miR-188 mimic and FOXL1 pcDNA3.1, respectively. 1: mimic-ctr+vector; 2: miR-188 + vector; 3: miR-188 + FOXL1. *p < 0.05, **p < 0.01.

**Figure 5:**
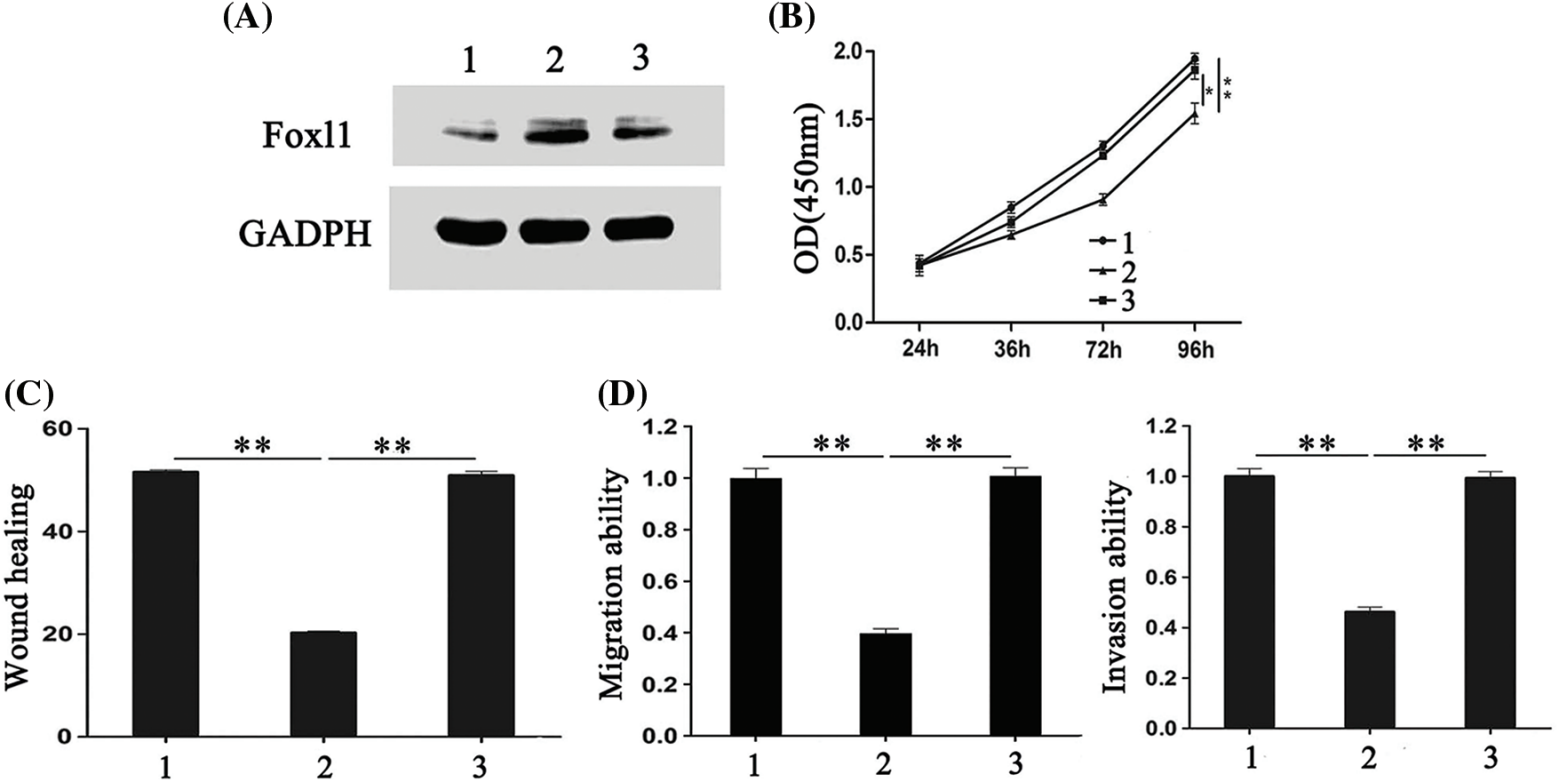
**The effects of miR-188 down-expression on colorectal cancer cells were rescued by FOXL1 down-expression.** (A) The expression of FOXL1 protein in LS174t cells were detected after transfection with miR-188 inhibitor and FOXL1 siRNA. (B-D) Cell proliferation, migration and invasion of LS174t was assessed after transfection of miR-188inhibitor and FOXL1 siRNA, respectively. 1: inhibitor-ctr+si-ctr; 2: miR-188-inhibitor + si-ctr; 3:miR-188-inhibitor + si-FOXL1. *p < 0.05, **p < 0.01.

To further explore whether FOXL1 mediated the effect of miR-188 on CRC cell proliferation, migration, and invasion, pcDNA-FOXL1 and miR-188 mimic or miR-ctr were co-transfected into SW480 cells, whereas FOXL1 siRNA and miR-188 inhibitor or miR-ctr were co-transfected into LS174t cells. The western blot results showed that the level of FOXL1 protein was decreased by miR-188 overexpression in SW480 cells, while overexpression of FOXL1 by pcDNA-FOXL1 abolished the suppression of FOXL1 caused by the miR-188 mimic (Fig. 4A). Further exploration suggested that overexpression of FOXL1 attenuated the suppressive effect of the miR-188 mimic on cell proliferation, migration, and invasion (Figs. 4B–4D). Western blot assays in LS174t cells demonstrated that the FOXL1 protein level was significantly enhanced by the miR-188 inhibitor, and knockdown of FOXL1 by siRNA attenuated this elevation (Fig. 5A). In LS174t cells, the increased cell proliferation, migration, and invasion caused by the miR-188 inhibitor were rescued by suppressing FOXL1 (Figs. 5B–5D). These results further confirmed our conclusion that miR-188 promoted cancer progression by directly targeting FOXL1 in CRC.

### Ectopic FOXL1 inhibited activation of the Wnt/β-catenin signaling pathway in CRC cells

**Figure 6:**
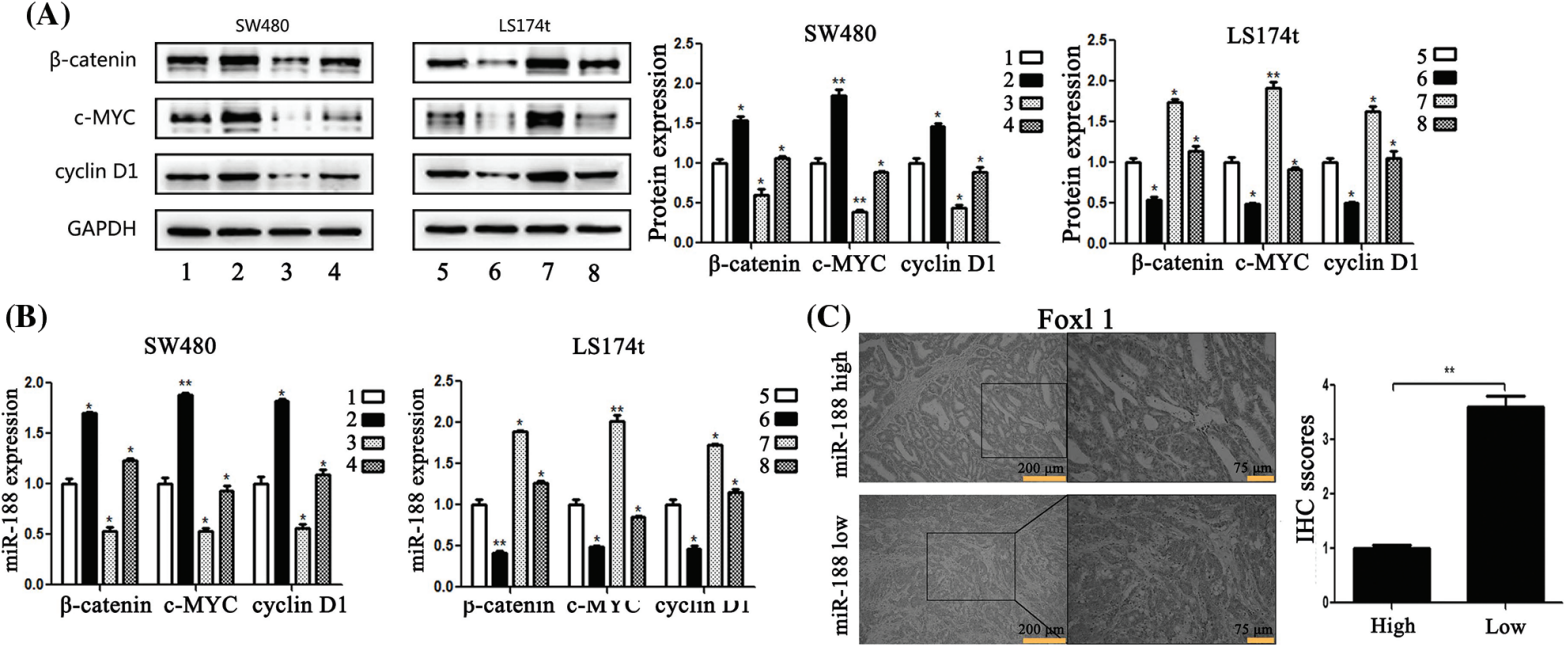
**MiR-188 regulated the expression of FOXL1 which affected the activation of the Wnt/β-catenin signaling pathway in colorectal cancer cells.** (A) Protein expression of β-catenin, c-MYC and cyclin D1 in both SW480 and LS174t cells was determined by western blot assays. (B) The results of real-time quantitative reverse transcription PCR. (C) Immunostaining of FOXL1 protein in colorectal cancer tissue samples. 1: mimic-ctr + vector; 2: miR-188 + vector; 3: miR-ctr+FOXL1; 4: miR-188+FOXL1; 5: inhibitor-ctr+si-ctr; 6: miR-188-inhibitor+si-ctr; 7: inhibitor+si-FOXL1; 8: miR-188-inhibitor + si-FOXL1. *p < 0.05, **p < 0.01.

To gain insight into the molecular mechanism underlying the effects of FOXL1 dysregulation in CRC cells, we investigated the effect of FOXL1 on the Wnt/β-catenin signaling pathway in SW480 and LS174t cells using qRT-PCR and western blotting. PcDNA-FOXL1 and miR-188 mimic or miR-ctr were co-transfected into SW480 cells, whereas si-FOXL1 and miR-188 inhibitor or miR-ctr were co-transfected into LS174t cells. Detection o western blot revealed that miR-188 mimic significantly increased the expression of β-catenin, c-Myc, and cyclin D1 proteins and these effects could abolished by the overexpression of FOXL1. There was a decrease of β-catenin, c-Myc, and cyclin D1 expression in pcDNA-FOXL1 transfected cells, and this impact could be rescued by the miR-188 mimic in SW480 cells. Opposite effects were observed in LS174t cells co-transfected with miR-188 inhibitor, si-FOXL1, and controls. miR-188 downregulation could dramatically reverse the stimulative effects of si-FOXL1 on β-catenin, c-Myc, and cyclin D1. Consistently, the suppressive effects of miR-188 knockdown on β-catenin, c-Myc, and cyclin D1 could also be reversed by silencing FOXL1 (Fig. 6A). Furthermore, detecting mRNA levels by qRT-PCR supported the same conclusion (Fig. 6B). These results suggested that FOXL1 mediated the promoting effects of miR-188 on cell proliferation, migration, and invasion through the Wnt/β-catenin signaling pathway in CRC.

## Discussion

As important members of the non-coding RNA family, miRNAs are associated with many physiological and pathological processes. Aberrantly expressed miRNAs can disrupt tightly regulated RNA networks in multiple kinds of cells, triggering the development of cancer and subsequent metastasis [16,17]. A few studies identified the clinical significance of miR-188 as a prognostic biomarker. miR-188 is downregulated in several diseases including hepatocellular carcinoma and prostate cancer by suppressing growth and metastasis of cancer cells [10,11]. Moreover, Ruedel et al. demonstrated that miR-188 expression correlated to the activation state of RASF and inhibited migration of those cells [18].

Some studies show that low miR-188 expression could be potentially used as a prognostic biomarker [19]. Here, we showed that miR-188 may exert a vital role in the tumorigenesis and progression of human colon cancer. We also investigated the role of miR-188 in tumorigenesis and surprisingly discovered that it was significantly increased in human CRC tissues and cell lines. By analyzing the expression of miR-188 in cancer and non-cancer tissues, we found that its overexpression was significantly correlated with tumor stage and might serve as an independent predictor for poor outcomes of CRC patients. In addition, it was elucidated that miR-188 promoted cell proliferation, invasion, and migration in CRC. According to the literature, miR-188 was aberrantly expressed in diverse types of diseases such as gastric cancer, thyroid carcinoma, and bladder cancer [20–22]. The exact role of miRNA variable in different tumor types or different micro-environment. For example, miR-215 acts as an oncogene in glioma and gastric cancer cells [23,24], but plays a tumor suppressor role in CRC [25]. Interestingly, it was reported that the specific miRNA in this study, miR-188, acted as an oncogene in gastric cancer but anti-cancer gene in breast cancer, which further indicated miR-188 may play multifaceted roles with different cancer cells specificity [22,26].

Importantly, there was a research found that miR-188 was down-regulated in CRC and it mediated the promoting tumorigenesis effects of LINC00668 in CRC cells, which indicated miR-188 might be an anti-cancer gene in CRC [27]. Herein we would like to discuss the divergence and potential reasons. Firstly, the research of Yan demonstrated that miR-188 was down-regulated in CRC tissues than normal tissues but our detection showed opposite results. In order to obtain the third parties’ verification, we plotted the TGCA data by using STARBASE 3.0. As showed in Fig. 1D, the results indicated that miR-188 was significantly up-regulated in CRC tissues than normal tissues, which was consistent with our results and not with the Yan’s. The different expression of miR-188 between two these two study may attribute to the sample size and should be verified in future study. Secondly, for the biological function of miR-188 in CRC, the research by Yan’s group did not explored the functional changes in CRC cells by over-expressing or down-regulating miR-188 purely and the effects of miR-188 were only speculated in the influences of LINC00668. As we known, the biological roles of miRNA might be affected by changes of micro-environment. We hold the opinion that it should be explored in exact micro-environment without other molecular influences. Thus we only changed the miR-188 in functional assays and demonstrated that miR-188 might play promoting roles in CRC. Last but not least, the previous study demonstrated that there was predicted binding site between miR-188 and LINC00668 by using STARBASE 3.0 and then verified. However, we failed to find the gene of LINC00668 in STARBASE 3.0 when we tried to discuss the divergence, which might attribute to the technical reasons. Hence, the difference between studies is interesting and important and it deserves further comprehensive study in the future.

MiRNAs normally cause translational repression by binding to the 3’-UTR of the mRNA of their target genes. As mentioned before, several target genes of miR-188 have been verified, including LAPDM4B and FGF5. Among potential targets of miR-188 provided by target predicting tools, FOXL1 attracted our attention because it was implicated in the regulation of epithelial cell proliferation in the gastrointestinal tract [12] and was found in several kinds of tumorous diseases. In addition, Perreault et al. found that FOXL1 was the first mesenchymal modifier of Min and played a key role in gastrointestinal tumorigenesis [28].

FOXL1 expression is downregulated in gastric cancer tissues and its expression significantly correlates with tumor stage, lymph node metastasis, and distant metastasis [29]. Furthermore, upregulation of FOXL1 greatly inhibits cell proliferation, migration, and invasion *in vitro*, and tumorigenicity of gallbladder cancer in nude mice [30]. FOXL1 was identified as a direct target of miR-188 by luciferase reporter assays. We found that miR-188 promoted proliferation, invasion, and migration of CRC cells through the downregulation of FOXL1 by directly binding to its 3’-UTR. The effect of its binding on suppressing FOXL1 was verified by detecting mRNA and protein levels. Our exploration is the first to uncover that mir-188 has a role as an oncogene in human CRC via suppressing the expression of FOXL1.

After identifying the inhibitory effect of miR-188 on FOXL1, we explored the molecular mechanism underlying how FOXL1 regulated downstream signaling and played a tumorigenic role in CRC. Wnt signaling is a critical pathway for regulating tissue development and adult tissue homeostasis, and β-catenin is a main downstream effector of the canonical Wnt signaling pathway [31]. After the Wnt pathway is activated, β-catenin is released from the “destruction complex” and translocates into the nucleus where it can activate the transcription of multiple target genes such as cyclin D1, c-Myc, and matrix metalloproteinases which are implicated in cell differentiation, proliferation, migration, and invasion [32–34]. However, aberrant activation of Wnt signaling will promote cell proliferation and enhance characteristics of the malignant phenotype in CRC, and is associated with poor prognosis of CRC patients [35–37].

The study of Perreault et al. indicated that FOXL1 might activate the Wnt/β-catenin pathway by increasing extracellular proteoglycans, which act as co-receptors for Wnt [12]. These researchers established that FOXL1 might be involved in the regulation of the Wnt/β-catenin pathway, providing a potential link to mesenchymal/epithelial cross-talk in the gastrointestinal tract. In the current study, we decided to explore the correlation between miR-188-mediated changes in FOXL1 and the Wnt/β-catenin signaling pathway. We observed that overexpression of FOXL1 significantly downregulated the expression of β-catenin, c-Myc, and cyclin D1 mRNA and protein in CRC cells. These changes could be retarded by upregulation of miR-188. Inversely, the stimulating effect of silencing FOXL1 on β-catenin, c-Myc, and cyclin D1 could be abolished by miR-188 knockdown. These findings strongly suggest that miR-188 regulated the proliferation, invasion, and migration of CRC cells through FOXL1 by influencing the Wnt/β-catenin signaling pathway. These were some study before illustrated the effects of miR-188 on Wnt/β-catenin signaling pathway. Yun et la found that miR-188 directly targeted PTEN to affect Wnt/β-catenin signaling in gastric cancer [38]. Yang et al demonstrated that miR-188 significantly promoted EMT by down-regulating DNA binding 4 through Wnt/β catenin signaling in retinoblastoma [39]. The difference in these studies indicated that miR-188 might regulated Wnt/β-catenin signaling through different molecules in different cancer types. Or the functions of miR-188 in Wnt/β-catenin signaling might not depend on single factor. Our study is the first research to verify the tumor-promoting role of miR-188 in CRC cells and validate that FOXL1 is the key component between miR-188 and Wnt/β-catenin signaling in CRC.

In summary, miR-188 was significantly upregulated in human CRC tissue samples and cell lines. MiR-188 promoted the proliferation, migration, and invasion of CRC cells through inhibiting the expression of FOXL1 and activating the Wnt/β-catenin signaling pathway. These results provide new insights into the diagnosis and treatment of CRC. Importantly, our findings indicate that miR-188/FOXL1/Wnt/β-catenin may serve as prospective therapeutic targets of CRC in the future.

## Data Availability

The data that support the findings of this study are available on request from the corresponding author.

## Abbreviations

miR-188: microRNA-188-5p
CRC: colorectal cancer
UTR: untranslated region
FOXL1: Forkhead Box L1
qRT-PCR: quantitative real-time polymerase chain reaction
siRNA: small interfering RNA
CCK8: cell counting kit 8
SEM: standard error of the mean
